# Assessing the Impact of 2D:4D Ratio on Oral Hygiene Performance and Gingival Health in Turkish Adults

**DOI:** 10.3290/j.ohpd.c_2767

**Published:** 2026-07-28

**Authors:** Ekin Beşiroğlu-Turgut, Gökçe Aykol-Şahin, Yiğit Cem Öğretmen

**Affiliations:** a Ekin Beşiroğlu-Turgut Dentist, Department of Periodontology, Faculty of Dentistry, Istanbul Okan University, Istanbul, Turkey. Conceived the research idea, analyzed the data, wrote the manuscript.; b Gökçe Aykol-Şahin Associate Professor, Department of Periodontology, Faculty of Dentistry, Istanbul Okan University, Istanbul, Turkey. Wrote the manuscript.; c Yiğit Cem Öğretmen PhD Candidate, Department of Periodontology, Faculty of Dentistry, Istanbul Okan University, Istanbul, Turkey. Collected and analyzed the data.

**Keywords:** dental hygiene, digit ratios, gingivitis, handedness, sex characteristics.

## Abstract

**Purpose:**

To investigate the relationship between the second-to-fourth digit ratio (2D:4D), a marker of prenatal hormone exposure, and oral hygiene performance and gingival health.

**Materials and Methods:**

A cross-sectional study was conducted with 126 adults aged 18–55 years (71 males, 55 females) at Istanbul Okan University’s Periodontology Clinic. Participants were classified by sex, hand dominance, and 2D:4D ratio (cut-off: 1). Oral hygiene was assessed using the Turesky Modification of the Quigley-Hein Plaque Index (QHPI) and Gingival Index (GI) at baseline and one week after standardised oral hygiene education. Finger lengths were measured to calculate 2D:4D. Statistical analyses included t-tests, repeated-measures ANOVA, Pearson’s correlation, and Bonferroni post-hoc tests (α=0.05).

**Results:**

No statistically significant difference in 2D:4D ratio was found between sexes (p=0.395) or hand dominance (p=0.730). QHPI and GI scores statistically significantly improved across all groups after education (p<0.001). No statistically significant association was found between 2D:4D ratios and changes in QHPI or GI. However, left-handed individuals showed statistically significantly lower QHPI scores at both time points (p< 0.05), and males with 2D:4D <1 reported more frequent brushing habits, though this was not reflected in clinical indices. The highest GI values were observed in males with 2D:4D ≥1, while the lowest were in females with 2D:4D <1.

**Conclusions:**

The 2D:4D ratio was not a reliable predictor of oral hygiene status or gingival health. However, handedness may influence oral hygiene performance. Standardized oral hygiene education led to comparable improvements regardless of biological variability.

The second-to-fourth digit ratio (2D:4D)—the ratio of the length of the index finger to that of the ring finger—has gained increasing attention as a non-invasive, stable biomarker of prenatal sex hormone exposure. This morphological trait is shaped during early fetal development and remains relatively constant from early childhood into adulthood. Its formation is governed by a shared genetic mechanism controlling both digit and gonadal development, particularly involving the Hox A and Hox D gene clusters.^15,23^ The 2D:4D ratio is believed to reflect the balance of prenatal androgens (especially testosterone) and estrogens. Lower ratios (more masculinized) indicate higher prenatal testosterone exposure, whereas higher ratios (more feminized) are associated with lower testosterone and higher estrogen levels. Typically, male fetuses—exposed to higher levels of testosterone—exhibit lower 2D:4D ratios than female fetuses.^23^

Research has linked variations in 2D:4D to a broad spectrum of behavioral traits, cognitive abilities, and health outcomes. Elevated prenatal testosterone, as indexed by a lower digit ratio, has been associated with male-typical traits such as increased aggression, enhanced visuospatial abilities, superior athletic performance, and higher mathematical proficiency. Conversely, higher 2D:4D ratios have been related to female-typical traits and increased risk for conditions such as obesity, cardiovascular disease, and infertility.^14,20^ These associations underscore the digit ratio’s potential as a window into the long-term developmental effects of prenatal hormonal environments.

In particular, 2D:4D has been shown to have strong associations with motor performance, especially skills that require fine motor coordination. Most studies in this area have focused on athletic populations, reporting that lower digit ratios correlate with better performance in sports requiring speed, strength, and coordination.^39^ Fink et al,^4^ studying right-handed Austrian children, observed that higher 2D:4D ratios were associated with better hand skill performance, particularly in males, suggesting a link between lower prenatal testosterone exposure and enhanced fine motor abilities. Similarly, Mathangi et al^24^ found gender-based differences in Indian children aged 8 to 12: males excelled in gross and fine motor speed, while females outperformed in precision-demanding tasks—highlighting both biological and functional dimensions of motor development.

The connection between prenatal androgen exposure and cerebral lateralization has also been the subject of longstanding theoretical debate. The Geschwind-Behan-Galaburda (GBG) hypothesis posits that higher prenatal testosterone levels may inhibit left-hemispheric development, leading to compensatory growth in the right hemisphere and an increased likelihood of left-handedness, language impairments, and stronger visuospatial skills.^7,8^ In contrast, Witelson and Nowakowski^40^ proposed that testosterone facilitates hemispheric specialization by pruning interhemispheric connections, thereby enhancing right-handedness, especially in males. Supporting these divergent models, Grimshaw et al^9^ found that girls with higher prenatal testosterone levels displayed stronger right-hand preference and greater left-hemisphere language dominance. More recently, Fink et al^4^ used digit ratio as a proxy for prenatal testosterone and observed that higher exposure may slow development in certain left-hemispheric regions, leading to improved left-hand motor skills, while lower testosterone levels were associated with better right-hand performance. Taken together, these findings point to a complex interplay between prenatal hormonal exposure, brain development, and motor functioning. However, while many studies have examined the role of 2D:4D in sports and cognition, its relationship to health-related behaviors involving motor skills remains underexplored. One such domain is oral hygiene, which requires sustained fine motor coordination, particularly in actions such as toothbrushing and flossing.^11^ Despite the clear motor demands of such behaviors, the potential connection between 2D:4D and oral hygiene has not yet been systematically investigated.

The present study aims to fill this gap by exploring the association between the 2D:4D digit ratio and oral hygiene practices. By integrating biological markers with behavioral outcomes, the research aims to provide novel insights into how prenatal hormonal influences may shape everyday self-care behaviors through their impact on motor skill development.

## MATERIALS AND METHODS 

### Study Design 

This cross-sectional study was conducted between September 2023 and May 2024 in the Periodontology Clinic of the Faculty of Dentistry at Istanbul Okan University. The study protocol was approved by the Ethics Committee of Istanbul Okan University (Decision no: 2023/165-01).

Participants who were over 18 years of age, had no psychological or neurological disorders, were able to cooperate during examination and procedures, were either university students or graduates, had at least 28 teeth to allow accurate scoring, had no fixed orthodontic appliances or crown prostheses, did not smoke, had not previously received oral hygiene education from a dental professional, and had a clinical diagnosis of gingivitis were included in the study. The sample size was calculated with G*Power 3: two-tail; α=0.05; power (1-β)=0.80.^6^ A total of 126 participants, aged between 18 and 56 years, who attended our Periodontology Clinic for a routine dental examination, agreed to participate in the study, and provided written informed consent were included in the study. To eliminate inter-examiner variability, all oral hygiene indices and digit measurements were performed by a single examiner (YCÖ). Before data collection, the examiner underwent standardized training and was evaluated for accuracy and reliability. The calibration process continued until reproducibility exceeded 90% for both measurement types.^17^

During the initial examination, socio-demographic data were recorded. To assess oral hygiene and gingival health status, the Turesky Modification of the Quigley-Hein Plaque Index^36^ (QHPI) and the Gingival Index^19^ (GI) were used, and the participants’ frequency of toothbrushing was also noted.

The QHPI was applied to all teeth except third molars, with both buccal and lingual surfaces examined. Dental plaque was disclosed using Mira-2-Ton Disclosing Solution (Hager & Werken; Duisburg, Germany). Scoring was between 0 and 5 (0: no plaque or debris present; 1: isolated flecks of plaque at the cervical margin of the tooth; 2: a thin, continuous band of plaque (up to 1 mm in width) at the cervical margin; 3: a band of plaque wider than 1 mm but covering less than one-third of the crown; 4: plaque covering at least one-third but less than two-thirds of the crown; 5: plaque covering two-thirds or more of the crown).^36^

The GI was used to evaluate gingival inflammation and was scored from 0 to 3 (0: healthy gingiva; 1: mild inflammation and edema, no bleeding on probing; 2: moderate inflammation and edema, bleeding on probing; 3: severe inflammation and tendency to spontaneous bleeding). Measurements were recorded at three sites – mesial, mid-buccal, and distal – on both the buccal and lingual surfaces of all teeth.^19^

The lengths of the second (index finger) and fourth (ring finger) digits of the right hand were measured on the ventral surface using a digital calliper, from the fingertip to the midpoint of the basal crease.^23^ The 2D:4D ratio was calculated by dividing the length of the second digit by the length of the fourth digit. To increase measurement reliability, three measurements were taken for each finger, and the average value was recorded. A cut-off value of 1 was used to assess the influence of prenatal androgens. Both male and female participants were categorized into two subgroups based on their 2D:4D ratios: those with a ratio ≥1 and those with a ratio <1.^22^

Following the initial clinical examination and data collection, all participants received standardized oral hygiene instructions from the same instructor, demonstrated on a dental model and practiced in front of a mirror, ensuring at least 15 min were dedicated to each patient for the sake of uniformity. Participants were instructed to brush their teeth twice daily for a minimum of 2 min utilizing the Modified Bass technique. Furthermore, depending on their individual embrasure dimensions, the use of dental floss or interdental brushes was recommended at least once daily. The second clinical examination was conducted one week later by the same investigator, during which both the QHPI and the GI were re-scored for each participant. Since changes associated with gingival inflammation can reverse within 7 days upon resumption of routine oral hygiene practices, the follow-up appointment was scheduled at one week.^13^

### Statistical Analysis

Descriptive statistics for the data were calculated and presented as mean ± SE. Prior to hypothesis testing, the data were assessed for compliance with parametric test assumptions: normality was evaluated using the Shapiro-Wilk test, and homogeneity of variances was examined using Levene’s test. Student’s t-test was employed to compare 2D:4D ratios based on gender and dominant hand. To investigate the effects of gender, dominant hand, and study groups on QHPI and GI measurements over time (day 0 and day 7), a two-way mixed design ANOVA was conducted for each factor using the general linear model technique for repeated measures. When significant main effects were identified within the model, Bonferroni post-hoc tests were applied for further analysis. Pearson correlation analysis was used to examine the strength and direction of the relationship between 2D:4D ratios and GI and QHPI measurements. A significance level of α=0.05 was adopted for all statistical evaluations. Statistical analyses were performed using the SPSS 30 software package.

## RESULTS

The study included 126 participants, comprising 71 males and 55 females. Participants’ ages ranged from 18 to 55 years, with a mean ± SD of 28.21 ± 0.82 years. The mean 2D:4D ratio was 0.98 ± 0.005 in females and 0.97 ± 0.004 in males; however, this difference was not statistically significant (p=0.395). Regarding hand dominance, 105 participants were right-handed and 21 were left-handed. The mean 2D:4D ratio was 0.977 ± 0.004 in right-handed individuals and 0.984 ± 0.01 in left-handed individuals, with no statistically significant difference observed (p=0.730).

There were no statistically significant differences in QHPI measurements between male and female participants at either day 0 or day 7 (p>0.05). Similarly, when participants were grouped according to sex and 2D:4D ratio, no statistically significant differences in QHPI scores were observed on either measurement day (p>0.05). However, when examining the effect of group assignment on QHPI measurements over time, a statistically significant overall reduction in QHPI scores was found across all groups from day 0 to day 7 (p<0.05). Although the largest decrease in QHPI scores was noted in the female group with a 2D:4D ratio ≥1, this reduction was not statistically different from the changes observed in the other groups (p=0.207) (Table 1).

**Table 1:** Comparison of QHPI scores on day 0 and day 7 according to the 2D:4D ratios in male and female participants

	**QHPI day 0**			**QHPI day 7**		p-value*		
Group	n	Mean± SE	SD	Mean± SE	SD	Group	Time	Group × Time
Female 2D:4D<1	46	2.897 ± 0.168 a	1.137	2.048 ± 0.128 b	0.865	0.322	<0.001	0.207
Female 2D:4D≥1	9	2.7 ± 0.202 a	0.607	1.876 ± 0.06 b	0.181			
Male 2D:4D<1	51	2.483 ± 0.143 a	1.021	1.835 ± 0.125 b	0.891			
Male 2D:4D≥1	20	2.467 ± 0.106 a	0.472	2.016 ± 0.18 b	0.804			
SD: standard deviation; SE: standard error. a,b: Different letters in the same row indicate statistically significant change over time (p<0.05). *Two-way mixed design ANOVA.								

No statistically significant difference was observed between male and female participants in GI scores measured on day 0 and day 7 (p>0.05). When participants were categorized by sex and 2D:4D ratio, GI scores decreased statistically significantly and similarly over time in all groups (p<0.05). Additionally, statistically significant differences were observed among the groups in terms of GI scores measured on both day 0 and day 7 (p<0.05). According to the results of multiple comparisons, the highest GI scores were observed at both timepoints in males with a 2D:4D ratio ≥1, while the lowest scores were found in females with a 2D:4D ratio <1. The difference between these two groups was statistically significant (p<0.05). A statistically significant difference was also found between males with 2D:4D <1 and those with 2D:4D ≥1, whereas no statistically significant difference was observed among the female subgroups (Table 2).

**Table 2:** Comparison of GI scores on day 0 and day 7 according to the 2D:4D ratios in male and female participants

				
								
								
					
					
					


When QHPI scores were examined based on hand dominance, a statistically significant difference was found between right- and left-handed individuals at both day 0 and day 7 (p<0.05). Right-handed participants exhibited statistically significantly higher QHPI scores at both time points compared to their left-handed counterparts (p<0.05) (Table 3). In contrast, no statistically significant differences were found between right- and left-handed participants in GI scores at either day 0 or day 7 (p>0.05) (Table 4).

**Table 3:** Comparison of QHPI scores on day 0 and day 7 based on the dominant hand

	**QHPI day 0**			**QHPI day 7**		p-value*		
Dominant hand	n	Mean± SE	SD	Mean± SE	SD	Dominant hand	Time	Sex × time
Right	105	2.738 ± 0.101 a,A	1.035	2.015 ± 0.082 b,A	0.843	0.014	<0.001	0.502
Left	21	2.191 ± 0.117a,B	0.534	1.588 ± 0.154 b,B	0.707			
SD: standard deviation; SE: standard error. Different lowercase letters in the same row indicate statistically significant change over time (p<0.05); Different uppercase letters in the same column indicate statistically significant differences between right and left dominant hands at each measurement time (p<0.05). *Two-way mixed design ANOVA.								

**Table 4:** Comparison of GI scores on day 0 and day 7 based on the dominant hand

	**GI day 0**			**GI day 7**		p-value*		
Dominant hand	n	Mean± SE	SD	Mean± SE	SD	Dominant hand	Time	Sex x time
Right	105	0.978 ± 0.035 a	0.357	0.785 ± 0.031 b	0.315	0.216	<0.001	0.258
Left	21	1.038 ± 0.076 a	0.349	0.909 ± 0.048 b	0.218			
SD: standard deviation; SE: standard error. Different lowercase letters in the same row indicate statistically significant change over time (p<0.05). *Two-way mixed design ANOVA.								

Among all groups, male with a 2D:4D ratio <1 were found to have a higher frequency of brushing their teeth twice a day or more. In contrast, both female subgroups exhibited a higher prevalence of brushing once a day or irregularly compared to males (Table 5).

**Table 5:** Brushing frequency according to the 2D:4D ratios in male and female participants

	**Brushing frequency**		
	Once a day/Irregularly	2 times a day or more	p-value*
Group	n (%)	n (%)	
Female 2D:4D<1	31 (67.4%) a	15 (18.8%) b	<0.001
Female 2D:4D>=1	7 (13.7%) a	2 (2.5%) b	
Male 2D:4D<1	6 (11.3%) a	45 (56.3%) b	
Male 2D:4D>=1	2 (3.9%) a	18 (22.5%) b	
Different letters within the same row indicate a statistically significant difference in column proportions for toothbrushing frequency within each study group (p<0.05). *Fisher-Freeman-Halton test.			

## DISCUSSION

The potential effect of the 2D:4D finger ratio on the oral hygiene level and toothbrushing habits of individuals was investigated in the present study. The 2D:4D ratios have been hypothesized to offer insight into a person’s prenatal life, providing information about their behavior, the likelihood of disease occurrence, intelligence, and abilities.^12,34,37^ The results of this study did not confirm previous findings that there is a significant sex difference in the 2D:4D ratio, which is a lower ratio found in males in the healthy population.^5,31^ This finding suggests that it may reflect sample-specific characteristics, methodological differences, or population variability. Therefore, its reliability as a consistent marker may be questionable. Nonetheless, categorizing participants according to high and low 2D:4D ratios in our study enabled us to assess the association between this potential biomarker of prenatal hormonal exposure and oral hygiene behaviors, considered separately from sex. Although the 2D:4D ratio has been mostly associated with motor skills, cognitive tendencies, and some personality traits in the existing literature, the power of this biomarker in predicting daily health behaviors remains unclear.^2,21^ In this context, the present study is one of the few to evaluate the effect of the 2D:4D ratio in the field of dentistry, and to our knowledge, none of them have focused on behavioral outcomes related to oral hygiene. The findings of this study indicate no statistically significant relationship between the 2D:4D ratio and the plaque index, gingival index, or frequency of toothbrushing. Similarly, previous research has reported no association between the 2D:4D ratio and periodontal health status.^29^ This finding suggests that the motor skill differences theoretically posited by Manning et al^21^ may not be directly reflected in habits such as oral hygiene. Supporting this notion, a study conducted among medical students found no statistically significant correlation between 2D:4D ratios and fine motor skills assessed by the Nine-Hole Peg Test (NHPT), further questioning the utility of this marker in predicting functional motor performance.^27^ It has also been suggested that the 2D:4D ratio may be more closely associated with multitasking performance requiring the integration of cognitive and motor processes, rather than with isolated motor skills.^16^ Several studies have found a statistically significant correlation between caries and the 2D:4D ratio. However, these studies have primarily focused on genetic taste perception and dietary habits, rather than evaluating oral hygiene levels.^30,33,38^

The beneficial effects of oral hygiene education and motivational interviewing on oral care behaviors are well-documented in the literature. Silk and Krok^35^ emphasized that individuals with suboptimal oral hygiene may require more frequent and tailored motivational interventions. Moreover, even a single session of motivational interviewing or oral hygiene education has been shown to produce significant improvements in plaque index and gingival index scores among individuals with gingivitis, underscoring the clinical relevance of brief interventions.^10^ In the present study, despite participants exhibiting a range of individual differences, including cognitive and hormonal variations, comparable improvements in plaque and gingival indices were observed following a single standardized oral hygiene education session. While individualized motivational approaches are often advocated,^18,32^ these findings imply that a certain level of educational attainment may enable individuals to achieve similar behavioral improvements in oral hygiene, irrespective of underlying gender or hormonal variability.

A further noteworthy finding in the present study was that plaque index values were statistically significantly lower in left-handed individuals. This finding corroborates the studies conducted by Ozden et al^28^ and Çiçek et al.^3^ They reported that left-handed individuals have better oral hygiene scores,^3,28^ which in one study were associated with lower halitosis values.^3^ Similar observations have also been reported in children aged 6–10 years, where left-handed brushing was associated with a higher prevalence of healthy gingiva.^25^ From a neuromotor perspective, hand preference is shaped by variations in speed and dexterity; the intrinsic strength and skill of the left hand primarily drive the adoption of left-hand dominance.^26^ Such differences could contribute to more effective toothbrushing in left-handed individuals; however, the underlying mechanisms remain unclear. Therefore, further studies incorporating objective, laboratory-based assessments of motor skills are needed to clarify this relationship.

Contrary to the findings reported in the extant literature, the present study revealed a statistically significantly higher proportion of males within the group that brushed their teeth twice a day or more. However, these findings were not reflected in the plaque index or gingival index scores. This inconsistency may be partly attributed to social desirability bias, whereby individuals tend to overreport positive health behaviors beyond their actual frequency in an effort to present a more favorable social image.^1^ A study reported that women generally exhibit better spontaneous oral hygiene behaviors; however, the gender difference in terms of relative improvement after treatment was not found to be significant.^1^ Moreover, some studies that report women as being more attentive to oral hygiene have not adequately accounted for educational level, which may act as a confounding variable.^41^ Therefore, our findings suggest that education, independent of gender, may enhance an individual’s oral care behaviours.

The strength of the current study was that participants were selected from a relatively homogeneous group of non-smokers who exhibited similar socioeconomic and educational levels, thereby eliminating other potential risk factors. However, the health literacy levels of individuals were not directly measured. This may also constitute a limitation, as it may influence individuals’ responses to oral hygiene education. Additionally, it may also be considered a limitation that some data, such as toothbrushing frequency, were obtained through self-reporting, which carries a risk of bias.

## CONCLUSIONS

The 2D:4D digit ratio did not appear to be a reliable predictor of specific biological traits and predicting complex health behaviors, such as daily oral hygiene. In contrast, handedness appeared to be associated with plaque accumulation, with left-handed individuals exhibiting lower plaque index scores, although the underlying mechanism remains unclear. Importantly, standardized oral hygiene education resulted in statistically significant improvements in plaque and gingival indices across all groups, regardless of sex or hormonal markers. These findings highlight the dominant role of modifiable behavioral interventions over inherent biological variability in oral hygiene outcomes. Future studies with larger samples and longitudinal designs are needed to further explore the role of individual characteristics such as handedness in oral health behaviors.
